# Calpain-6 Deficiency Promotes Skeletal Muscle Development and Regeneration

**DOI:** 10.1371/journal.pgen.1003668

**Published:** 2013-08-01

**Authors:** Kazuo Tonami, Shoji Hata, Koichi Ojima, Yasuko Ono, Yukiko Kurihara, Tomokazu Amano, Takahiro Sato, Yumiko Kawamura, Hiroki Kurihara, Hiroyuki Sorimachi

**Affiliations:** 1 Calpain Project, Department of Advanced Science for Biomolecules, Tokyo Metropolitan Institute of Medical Science, Kamikitazawa, Setagaya-ku, Tokyo, Japan; 2 Department of Physiological Chemistry and Metabolism, Graduate School of Medicine, The University of Tokyo, Hongo, Bunkyo-ku, Tokyo, Japan; 3 Animal Products Research Division, Institute of Livestock and Grassland Science, National Agriculture and Food Research Organization, Ikenodai, Tsukuba, Ibaraki, Japan; The Jackson Laboratory, United States of America

## Abstract

Calpains are Ca^2+^-dependent modulator Cys proteases that have a variety of functions in almost all eukaryotes. There are more than 10 well-conserved mammalian calpains, among which eutherian calpain-6 (CAPN6) is unique in that it has amino acid substitutions at the active-site Cys residue (to Lys in humans), strongly suggesting a loss of proteolytic activity. CAPN6 is expressed predominantly in embryonic muscles, placenta, and several cultured cell lines. We previously reported that CAPN6 is involved in regulating microtubule dynamics and actin reorganization in cultured cells. The physiological functions of CAPN6, however, are still unclear. Here, to elucidate CAPN6's *in vivo* roles, we generated *Capn6*-deficient mice, in which a *lacZ* expression cassette was integrated into the *Capn6* gene. These *Capn6*-deficient mouse embryos expressed *lacZ* predominantly in skeletal muscles, as well as in cartilage and the heart. Histological and biochemical analyses showed that the CAPN6 deficiency promoted the development of embryonic skeletal muscle. In primary cultured skeletal muscle cells that were induced to differentiate into myotubes, *Capn6* expression was detected in skeletal myocytes, and *Capn6*-deficient cultures showed increased differentiation. Furthermore, we found that CAPN6 was expressed in the regenerating skeletal muscles of adult mice after cardiotoxin-induced degeneration. In this experimental system, *Capn6*-deficient mice exhibited more advanced skeletal-muscle regeneration than heterozygotes or wild-type mice at the same time point. These results collectively showed that a loss of CAPN6 promotes skeletal muscle differentiation during both development and regeneration, suggesting a novel physiological function of CAPN6 as a suppressor of skeletal muscle differentiation.

## Author Summary

The calpains arose evolutionarily as a family of Ca^2+^-regulated intracellular proteases with divergent structures and functions. Unique among mammalian calpains, calpain-6 is “non-proteolytic”, and its physiological role has remained unknown. In this study, using *Capn6* knock-out mice, we discovered that Calpain-6 is involved in skeletal muscle development and regeneration *in vivo*. Calpain-6 was predominantly expressed in embryonic muscles and bone cartilage. *Capn6* knock-out promoted skeletal-muscle development as a result of increased progenitor cell differentiation. Furthermore, calpain-6 was detected in regenerating skeletal muscles, and suppressed this process. These results indicate that mammalian calpain-6 is a suppressive modulator for skeletal muscle differentiation and growth. Our findings indicate that calpain-6 might serve as a therapeutic target for muscular dystrophy/atrophy or as a useful tool in tissue engineering. For calpain studies, the physiological non-proteolytic role of calpain-6 may shed light on the elusive calpain functions that are independent of their well-studied proteolytic activities. In addition, as this structurally non-proteolytic feature is unique to the calpain-6 of placental mammals among vertebrates, our mutant mice might provide insight into the relationship between molecular and biological evolution.

## Introduction

Calpains (Clan CA-C2, EC 3.4.22.17) constitute a family of intracellular Ca^2+^-regulated cysteine proteases found in almost all eukaryotes and a few bacteria [1], [2]. They play indispensable roles in various biological processes, including cell migration, apoptosis, platelet aggregation, and myoblast fusion, through the limited proteolytic cleavage of diverse substrates [3]–[6]. The importance of calpains' physiological roles in mammals is revealed by the various phenotypes caused by calpain deficiencies, including embryonic lethality (in *Capn2*- or *Capns1*-deficient mice) [7]–[10], muscular dystrophies (human/mouse *CAPN3*/*Capn3*) [11]–[13], gastropathy (mouse *Capn8* and *Capn9*) [14], and vitreoretinopathy (human *CAPN5*) [15]. In addition, improper calpain activation can exacerbate a disorder or even cause death [16]–[18]. However, most *in vivo* functions of the calpains remain unclear.

Humans have 15 calpain genes that encode a calpain-like protease (CysPc) domain [19], [20] and are divided into subfamilies according to their domain structures. Calpains with a domain structure similar to that of CAPN1[μCL] and CAPN2[mCL] are termed “classical” calpains. They contain C2L (C2-domain-like) and PEF (penta-EF-hand) domains in addition to the CysPc domain. The “non-classical” calpains contain only the C2L or the PEF domain, or neither, and are subclassified accordingly. Of the human calpains, nine are classical (CAPN1-3, 8, 9, 11-14) and six are non-classical (CAPN5-7, 10, 15, 16).

Calpains are also categorized according to their tissue/organ distribution. Some human calpains are ubiquitously expressed (CAPN1, 2, 5, 7, 10, 13-16), whereas others are expressed only in specific tissues or organs (*e.g.*, CAPN3 in skeletal muscle, CAPN8/9 in gastrointestinal tracts, and CAPN11 in testis). It is widely assumed that the ubiquitous calpains play a fundamental role in all cells, while the tissue-specific calpains have tissue-specific roles [21].

The *Capn5* and *Capn6* genes were identified as orthologs of *tra-3*, a nematode gene for a sex-determination factor, TRA-3 [22], [23]. The encoded two mammalian TRA-3 orthologs, CAPN5 (also called hTRA-3) and CAPN6, share more than 30% amino acid (aa) identity with TRA-3. These calpains have a C2 (not C2L) domain at the C-terminus, *i.e.*, a “CysPc-C2L-C2” domain structure. CAPN5 has a Ca^2+^-dependent autolytic activity, is sensitive to several calpain inhibitors [24], and is expressed at varying levels in almost all tissues [25]. It is expressed by a subset of T cells, but the analysis of *Capn5^−/−^* mice showed that it is not required for development [26]. On the other hand, eutherian CAPN6 has a naturally occurring aa substitution at the active site Cys residue (Lys81 instead of Cys in human and mouse CAPN6), probably indicating a lack of proteolytic activity [25]. In terms of molecular evolution, metatherian (marsupial) and avian CAPN6 retain the active-site triad residues Cys-His-Asn, and frogs and fish have three TRA-3 homologs with conserved active-site residues.

Mammalian CAPN6 is predominantly expressed in embryonic muscles, placenta [27], and several cultured cell lines [28]. Using cultured cells, we previously found that CAPN6 regulates microtubule dynamics and actin reorganization by modulating the activity of a small G-protein [28], [29]. The function of CAPN6 *in vivo*, however, is still unclear. In this study, we examined CAPN6's physiological role by analyzing *Capn6*-deficient mice. To our surprise, the *Capn6*-deficient mice displayed precocious development of embryonic skeletal muscle. We also found that CAPN6 was expressed in regenerating skeletal muscle in adulthood, and that *Capn6* disruption promoted regeneration in cardiotoxin-injected skeletal muscle. Our results showed that a loss of CAPN6 promotes skeletal muscle differentiation during mouse development and regeneration, and suggest a novel physiological function for CAPN6 in suppressing differentiation in skeletal muscle.

## Results

### CAPN6 was successfully knocked-out in *Capn6-lacZ*-knock-in mice

**Figure 1:**
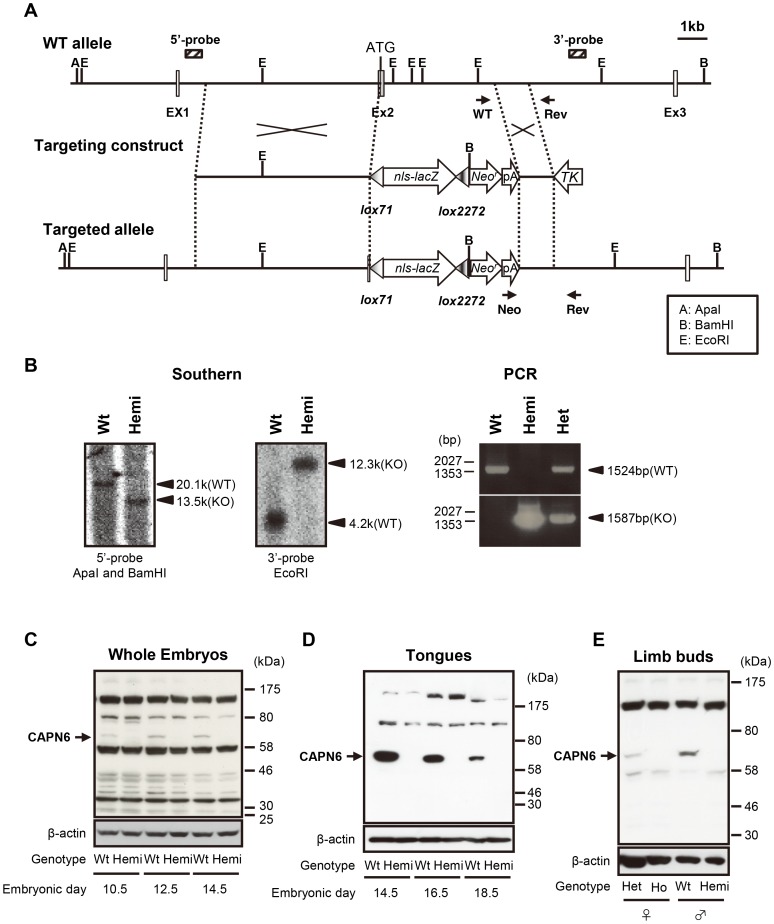
Targeted disruption of the *Capn6* gene. (A) Schematic representation of the targeting strategy used to knock-in an *nls-lacZ* cassette into the mouse *Capn6* locus. Exons 1 to 3 are indicated by open boxes with exon numbers. The 5′- and 3′-probes for Southern blotting are shown as hatched boxes. PCR primer positions for genotyping are indicated by arrows (see Table S1 for primers). Neo^r^, neomycin-resistance gene; pA, poly A tail; TK, thymidine kinase; A, *ApaI*; B, *BamHI*; E, *EcoRI*. (B) Southern blot (left) and PCR (right) analyses of genomic DNA extracted from mouse tails. The bands of the Southern blot represent *ApaI/BamHI*- or *EcoRI*-digested genomic DNA from wild-type and *Capn6^lacZ/Y^* mice, probed with the 5′- or 3′-probe. (C–E) Western blot analyses confirming the absence of CAPN6 in *Capn6*-deficient mice. Total lysates were obtained from E10.5, E12.5, and E14.5 whole embryos (C), the tongue of E14.5, E16.5, and E18.5 embryos (D), and limb buds of E13.5 embryos (E). A band of around 70 kDa (indicated by arrows) was diminished in the *Capn6*-deficient embryos and was presumed to be CAPN6, and all the other bands were considered to be non-specific. Wt, *Capn6^+/Y^* (♂); Hemi, *Capn6^lacZ/Y^* (♂); Het, *Capn6^lacZ/+^* (♀); Ho, *Capn6^lacZ/lacZ^* (♀).

We disrupted the mouse *Capn6* locus by replacing the protein-coding sequence in exon 2 with an *nls-lacZ/PGKneo* cassette (Figure 1A), and obtained *Capn6^lacZ/+^* heterozygous mice. The heterozygotes, which were all female, as *Capn6* is on the X-chromosome, appeared normal and were fertile. *Capn6*-deficient (*Capn6^lacZ/Y^* male or *Capn6^lacZ/lacZ^* female) mice were then obtained by intercrossing *Capn6^lacZ/+^* females with wild-type or *Capn6^lacZ/Y^* male mice, respectively.

To confirm the absence of CAPN6 protein, whole embryos (at embryonic day 10.5 [E10.5], E12.5, and E14.5) were chosen for western blot analysis, because *Capn6* mRNA is reported to be expressed during embryogenesis [27], [28]. As shown in Figure 1C, in wild-type embryo lysate, an anti-CAPN6 antibody detected the expected 74-kDa band, and this band was absent in lysates of *Capn6^lacZ/Y^* mice. *Capn6^lacZ/lacZ^* mice gave essentially the same results (data not shown; see below and Figure 1E). These results indicated that the CAPN6 protein is present in wild-type mouse embryos, and that at least full-length CAPN6 was lost in the *Capn6*-deficient mice. The CAPN6 bands were most clearly detected in the lysates from the embryonic tongue and limb buds (Figure 1D and E), which are rich in skeletal muscle. Notably, the signal intensities of the tongue CAPN6 were E14.5>E16.5>E18.5 (Figure 1D).

The CAPN6 signal in *Capn6^lacZ/+^* (♀) embryos was weaker than that in *Capn6^+/Y^* (♂) embryos (Figure 1E), even though both possessed one *Capn6* gene. This was probably because the *Capn6* expression was partly suppressed by X-chromosome inactivation in the female mice, as with many other X-linked genes [30].

### *Capn6* is expressed in embryonic muscles and cartilages

**Figure 2:**
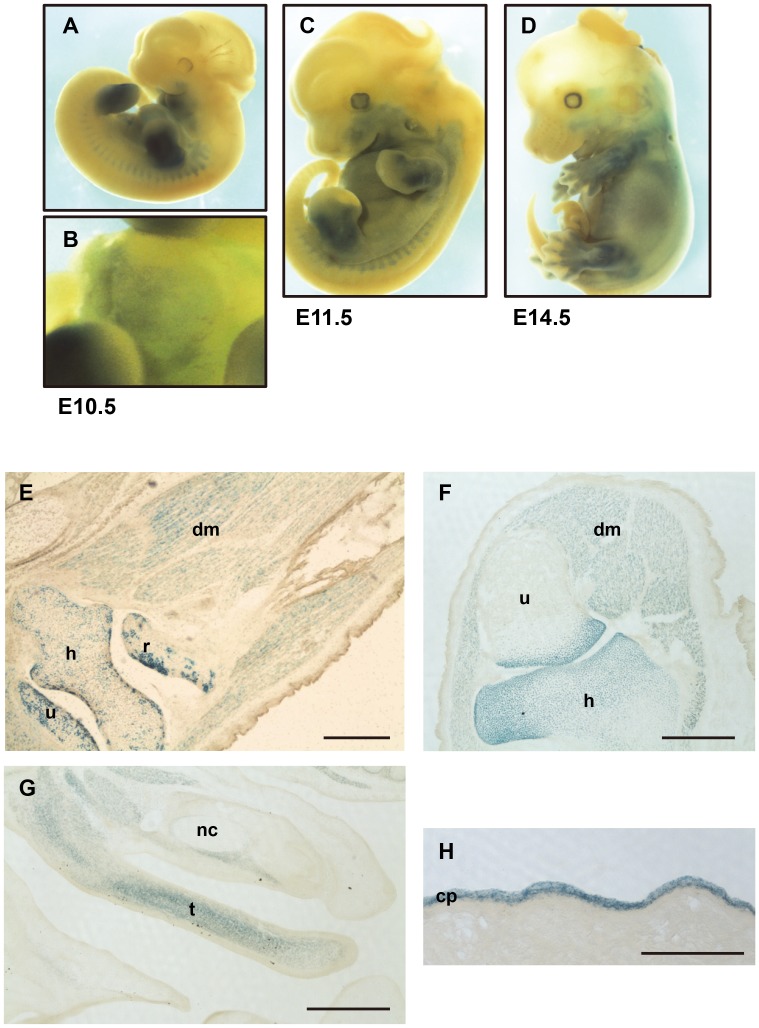
Expression of the *Capn6^lacZ^* allele. (A–D) Lateral views of *Capn6^lacZ/+^* (♀) whole embryos stained with X-gal at E10.5 (A), E11.5 (C), and E14.5 (D), and the magnified image of the heart field in A (B). (E–G) Frozen sections of *Capn6^lacZ/+^* (♀) or *Capn6^lacZ/lacZ^* (♀) embryos stained with X-gal. Coronal sections of the glenohumeral joint region of an E16.5 *Capn6^lacZ/+^* (E) and *Capn6^lacZ/lacZ^* (F) embryo, and sagittal section of the tongue primordium region of an E14.5 *Capn6^lacZ/lacZ^* embryo (G). (H) X-gal staining of *Capn6^lacZ/lacZ^* (♀) placenta (day 10.5 of gestation). β-Gal expression was found only in the chorionic plate (cp) of the placenta. dm, deltoid muscle; h, humerus; nc, nasal cavity; r, radius; t, tongue; u, ulna. Scale bars: 500 µm.

In our constructs, the null allele, *Capn6^lacZ^*, expressed ß-galactosidase (ß-Gal) in the cells where *Capn6* is transcribed in wild-type mice. To verify that the expression of *lacZ* reflected that of authentic *Capn6*, the ß-Gal activity was examined in whole-mount E10.5 *Capn6^lacZ/+^* embryos. As shown in Figures 2A and 2B, the mandibular arch, limb buds, somites, and heart were stained, consistent with the known *Capn6* expression pattern [27], [28]. At later stages (E11.5 and E14.5), ß-Gal was broadly expressed in the embryo (Figure 2C and D). Cryosections of E16.5 *Capn6^lacZ/+^* embryos showed ß-Gal expression in all the skeletal muscles and bone cartilage throughout the embryonic body (Figure 2E).

*Capn6^lacZ/lacZ^* (Figures 2F, G, and S1A–C) and *Capn6^lacZ/Y^* (data not shown) embryos, in which all the *Capn6* locus-active cells expressed *lacZ* and no *Capn6*, gave essentially identical results: ß-Gal expression was observed in all muscles, cartilages, and bones at the epiphysis, throughout the craniofacial area and trunk. No obviously abnormal morphology was observed in these regions. These *Capn6*-deficient mice were born healthily and grew to adulthood without any apparent abnormality. In adult mice, only the placenta showed ß-Gal expression (Figure 2H).

### *Capn6*-deficient embryos exhibit advanced muscular development

**Figure 3:**
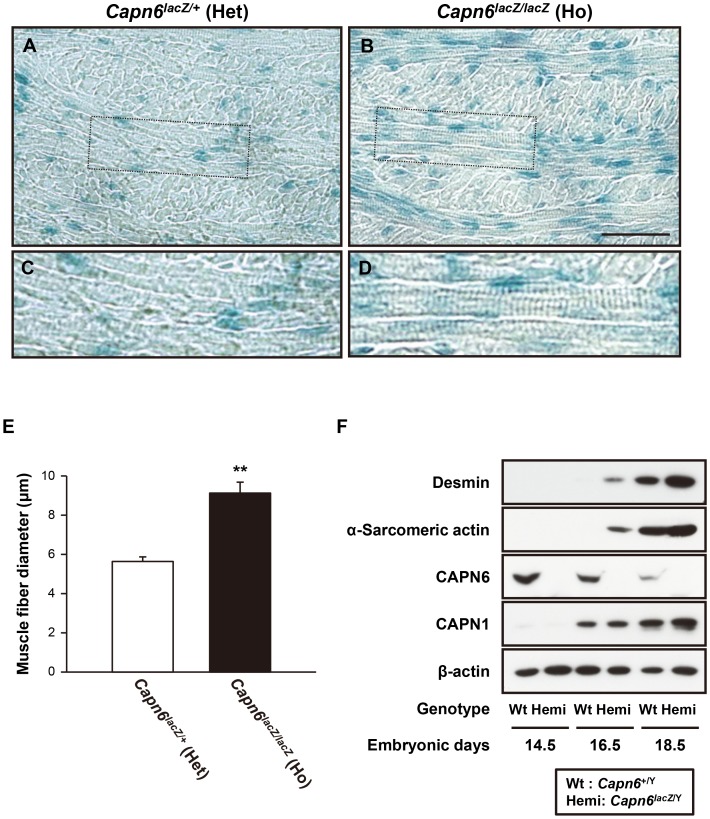
Advanced skeletal muscle development in *Capn6*-deficient embryos. (A–D) X-gal-stained coronal sections of the tongue of an E16.5 *Capn6^lacZ/+^* (A, C) and *Capn6^lacZ/lacZ^* (B, D) embryo. The boxed areas in A and B are enlarged in C and D, respectively. Scale bar: 50 µm. (E) The average diameter of tongue muscle fibers was measured in the anterior region (the areas in boxes a′ and b′ in Figure S2) of coronal sections. The average diameter was significantly larger in *Capn6^lacZ/lacZ^* (9.13 [mean]±0.56 [s.e.m.] µm, n = 5) than in *Capn6^lacZ/+^* (5.64±0.23 µm, n = 5). **, *P*<0.01 by Student's t-test. (F) Western blot analysis of the right limbs of E14.5, E16.5, and E18.5 embryos using antibodies against desmin, α-sarcomeric actin, CAPN6, CAPN1, and β-actin. Blotting for β-actin served as an internal control. Wt, *Capn6^+/Y^* (♂); Hemi, *Capn6^lacZ/Y^* (♂); Het, *Capn6^lacZ/+^* (♀); Ho, *Capn6^lacZ/lacZ^* (♀).

Given the high levels of *Capn6* expression in embryonic skeletal muscles, we focused on the physiological relevance of CAPN6 in skeletal muscle development. First, the morphology of the E16.5 tongue was compared between *Capn6^lacZ/+^* and *Capn6^lacZ/lacZ^* mice. As shown in Figures 3A–D, more advanced tube formation and striated structures were observed in the tongue muscle of *Capn6^lacZ/lacZ^* embryos than in their *Capn6^lacZ/+^* littermates. In addition, the diameters of the tongue muscle fibers in the *Capn6^lacZ/lacZ^* mice were significantly larger than in their *Capn6^lacZ/+^* littermates (Figures 3E and S2). To confirm these differences in skeletal muscle morphology, we examined the expression of desmin and α-sarcomeric actin, which are cytoskeletal components of skeletal muscle that play crucial roles in its development [31], [32]. Western blot analysis of embryonic limb-bud lysates showed that the expressions of desmin and α-sarcomeric actin increased with development, and that they were stronger in *Capn6^lacZ/Y^* embryos than in their *Capn6^+/Y^* littermates at E16.5 and E18.5 (Figure 3F). These results indicated that the deletion of CAPN6 advances the progression of skeletal muscle development during embryogenesis.

### Lack of CAPN6 promotes the differentiation of skeletal muscle progenitor cells

**Figure 4:**
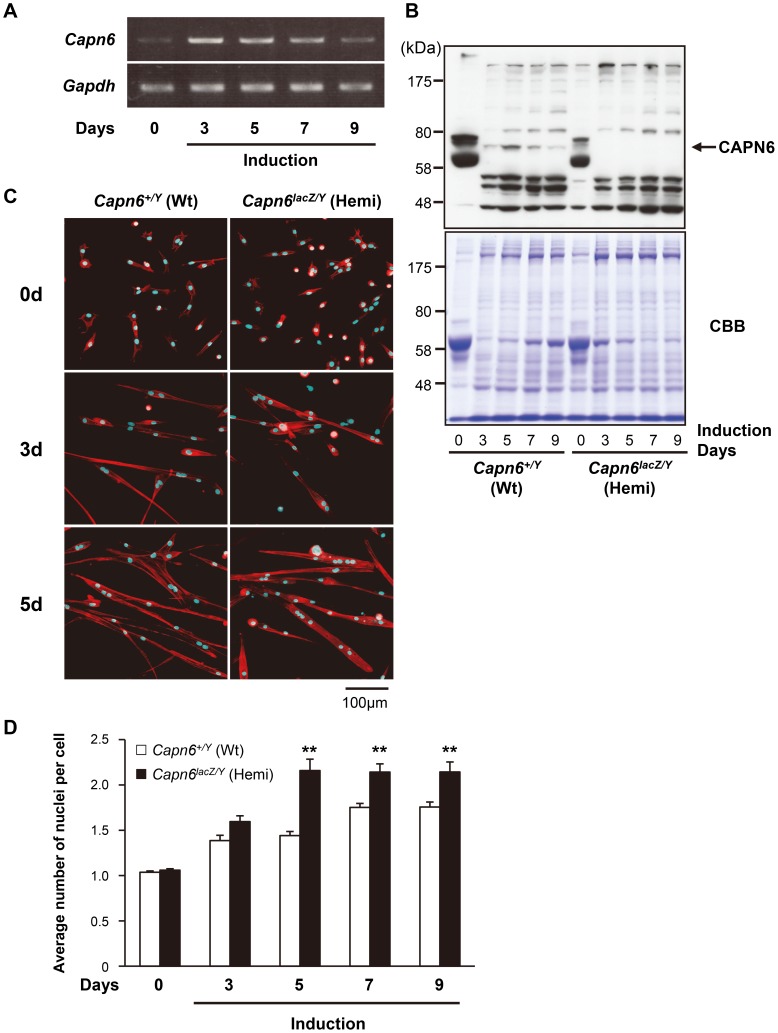
Increased differentiation of *Capn6*-deficient skm-primary cultured cells. (A) *Capn6* and *Gapdh* (internal control) mRNA was detected by PCR during the differentiation of skm-primary cultured cells. (B) Western blot analysis confirming the CAPN6 expression in skm-primary cultured cells. A band was detected around 70 kDa (indicated by arrow) in differentiating cells that was lost in *Capn6*-deficient cells. Thus, this 70-kDa band was presumed to be CAPN6, and all the other bands were considered to be non-specific. (C) Confocal images of skm-primary cultured cells. The cells were established from the skeletal muscles of 7-wk-old *Capn6^+/Y^* and *Capn6^lacZ/Y^* mice, and incubated in horse serum-containing medium for the indicated number of days to induce myotube differentiation. The nuclei and actin filaments of differentiated cells were visualized with DAPI (blue) and rhodamine-labeled phalloidin (red), respectively. Scale bar: 100 µm. (D) Average number of nuclei per cell in *Capn6^+/Y^* and *Capn6^lacZ/Y^* skm-primary cultured cells. Data are the mean values from three experiments (±s.e.m.), and 100 cells were counted in each experiment. The number of nuclei per cell was significantly greater in *Capn6^lacZ/Y^* than in *Capn6^+/Y^* after five days of differentiation (*Capn6^lacZ/Y^* vs *Capn6^+/Y^* are [2.16 [mean]±0.13 [s.e.m.] vs 1.44±0.04], [2.14±0.09 vs 1.75±0.04], and [2.14±0.11 vs 1.76±0.05] at 5, 7, and 9 days after induction, respectively). **, *P*<0.01 by Student's t-test. Wt, *Capn6^+/Y^* (♂); Hemi, *Capn6^lacZ/Y^* (♂).

To examine how the loss of CAPN6 promotes skeletal muscle development, we observed the differentiation of primary cultured skeletal muscle (skm-primary) cells from 7-week (wk) old *Capn6^+/Y^* and *Capn6^lacZ/Y^* littermates. The *Capn6^+/Y^* skm-primary cells were induced to differentiate into myotubes with horse-serum-containing medium. In these cultures, *Capn6* mRNA was detected in the differentiating myocytes as well as in undifferentiated skm-primary cells (Figure 4A). The CAPN6 protein was detected in the differentiating myocytes (Figure 4B). Its expression was upregulated after the induction of differentiation, and then gradually decreased.

Next, the differentiation of skm-primary cells was compared between *Capn6^+/Y^* and *Capn6^lacZ/Y^* littermates. DAPI staining demonstrated that the average number of nuclei in each cell derived from the *Capn6^lacZ/Y^* mice was greater than in their *Capn6^+/Y^* littermates 5–9 days after induction (Figure 4C and D). These results indicate that, in myotube differentiation, the skm-primary cells from *Capn6^lacZ/Y^* mice underwent fusion more rapidly than did those from *Capn6^+/Y^* mice. In other words, the disruption of CAPN6 promotes the differentiation of myocytes to myotubes, suggesting that CAPN6 acts as a suppressor of skeletal muscle differentiation.

### CAPN6 is involved in muscle regeneration

**Figure 5:**
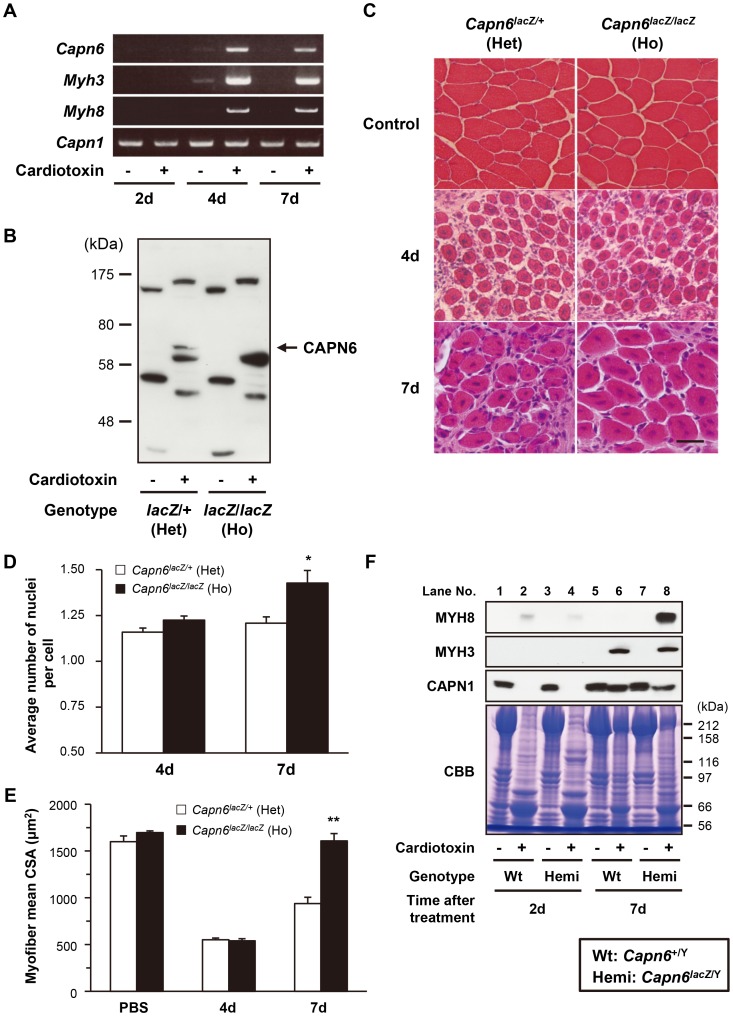
CAPN6 is expressed in regenerating skeletal muscles and suppresses regeneration. (A) RT-PCR with total RNAs extracted from skeletal muscles of 7-wk-old wild-type mice 2 d, 4 d, and 7 d after cardiotoxin or PBS (control) injection. The PCR bands of *Capn6* were amplified with the primers that detect full-length *Capn6* mRNA. *Myh3* and *Myh8* mRNA was amplified to confirm that skeletal muscles successfully regenerated after their cardiotoxin-induced degeneration; *Capn1* mRNA was detected as an internal control. (B) Western blot analysis using antibodies against CAPN6, of skeletal muscle lysates of 7-wk-old *Capn6^lacZ/+^* and *Capn6^lacZ/lacZ^* mice, 5 days after cardiotoxin or PBS (control) injection. The specific CAPN6 band, indicated by an arrow, was detected around 70 kDa, and was not detected in *Capn6^lacZ/lacZ^* mice. (C–E) Histological analysis of the TA muscles of 7-wk-old *Capn6^lacZ/+^* and *Capn6^lacZ/lacZ^* mice after a cardiotoxin or PBS (control) injection. Regenerating skeletal muscles with central nuclei were observed 4 d after the cardiotoxin injection. Remarkable degeneration was not observed in TA muscles at 4d after a PBS injection (shown as control) (C). Scale bar: 50 µm. Seven days after the injection, the average number of nuclei per cell was significantly greater in *Capn6^lacZ/lacZ^* (1.43 [mean]±0.06 [s.e.m.]) than in *Capn6^lacZ/+^* (1.21±0.03) (D), and the mean CSA of the regenerating muscle myofibers was also significantly greater in *Capn6^lacZ/lacZ^* (1608.0 [mean]±78.5 [s.e.m.] µm) than in *Capn6^lacZ/+^* (938.0±67.2 µm) (E). *, *P*<0.05; **, *P*<0.01 by Student's t-test. Each value is the mean from five experiments, and 50 cells were counted in each experiment. (F) Western blot analysis with skeletal muscle lysates of 7-wk-old *Capn6^lacZ/Y^* and *Capn6^+/Y^* mice 2d and 7 d after cardiotoxin or PBS (control) injection using antibodies against MYH3, MYH8 and CAPN1. CBB staining was used as an internal control. Wt, *Capn6^+/Y^* (♂); Hemi, *Capn6^lacZ/Y^* (♂); Het, *Capn6^lacZ/+^* (♀); Ho, *Capn6^lacZ/lacZ^* (♀).

The above experiments using skm-primary cells suggested that CAPN6 might be involved in skeletal muscle regeneration as well as its development. To examine skeletal muscle regeneration in adult mice, cardiotoxin was injected into the mouse tibialis anterior (TA) muscle. Cardiotoxin is a snake venom that selectively causes myofiber degeneration, but leaves nerves, blood vessels, and satellite cells morphologically intact [33]. Two days after the cardiotoxin injection, normal myofibers distinguished by marginal nuclei (shown as in the controls in Figure 5C) were diminished (data not shown). Then, 4d after the injection, the expression of skeletal muscle embryonic and skeletal muscle perinatal myosin heavy chain genes (*Myh3* and *Myh8*, respectively, in Figure 5A) and the cells' central nucleation (Figure 5C) showed that the degenerated TA skeletal muscle had begun to regenerate.

During this process, we examined *Capn6* expression by RT-PCR, using PBS-injected TA muscle as a control. *Capn6* mRNA was detected after 4d, similar to *Myh3* and *Myh8*, when muscle regeneration was taking place (Figure 5A). The CAPN6 protein was also detected in the regenerating muscle by western blot analysis (Figure 5B). Notably, histological analysis showed more nuclei in each myofiber in the regenerating skeletal muscles of the *Capn6^lacZ/lacZ^* mice than in the *Capn6^lacZ/+^* mice (Figure 5C and D). Furthermore, the cross-sectional area (CSA) of the regenerating skeletal muscles of the *Capn6^lacZ/lacZ^* mice was significantly larger than that of the *Capn6^lacZ/+^* mice (Figure 5C and E). Consistent with these observations, the expression of MYH8 was also higher at 7 days after cardiotoxin injection in the regenerating *Capn6^lacZ/Y^* muscles than the *Capn6^+/Y^* muscles (Figure 5F). To our surprise, the expression of CAPN1 was reciprocally suppressed with significance (*P* = 0.019; n = 3). These results indicated that the loss of CAPN6 promotes both the regeneration of skeletal muscle and its development.

## Discussion

In this study, we have shown a physiological function for CAPN6 for the first time: CAPN6 is involved in the suppressive modulation of skeletal muscle development and regeneration. This is the second reported calpain gene (after *CAPN3*/*Capn3*) to be genetically shown to be responsible for a skeletal muscle phenotype [12], [13], [34].

What is the physiological relevance of CAPN6's function as a skeletal muscle growth suppressor like myostatin and muscle RING-finger proteins (MuRFs) [35]–[37]? One possible function of CAPN6 is to restrict the embryo size of eutherians, because an embryo that is too large could harm the mother's body (known as “maternal-fetal conflict” [38]). This idea is compatible with the uniquely eutherian non-proteolytic character of CAPN6. During embryogenesis, CAPN6 is expressed not only in skeletal muscles but also in cartilage, the mandibular arch, somites, and the myocardium from early (E9.5) to late (E18.5) stages. Therefore, it is likely that CAPN6 also functions as a growth suppressor in tissues other than skeletal muscles. The role of CAPN6, however, must be accessory and/or stress-responsive, because the CAPN6 deficiency caused milder phenotype in the skeletal muscle systems under normal conditions than those of myostatin- or MuRFs-deficient mice [36], [39].

What is the molecular mechanism underlying CAPN6's action? We previously showed that CAPN6 modulates lamellipodial formation and cell motility by stabilizing microtubules and regulating Rac1 activity [28], [29]. Microtubule organization and the activity of small G-proteins are also crucial regulators of skeletal myogenesis [40], [41]. Thus, our collective results suggest that CAPN6 suppresses skeletal muscle differentiation and growth by modulating basal cellular functions such as cell division, growth, and cell migration through cytoskeletal reorganization. Molecules and a mechanism involved in these phenomena, however, may be different between skeletal muscles and cultured fibroblasts. It has been discussed that CAPN6 may antagonize other calpains to protect their substrates from cleavage [23], [27], as in the case of cFLIP, which is a protease-deficient caspase homolog that functions as an apoptosis inhibitor [42]. These points should be clarified in detail in future studies.

The changes in the level of another muscle-differentiation-related calpain, CAPN1, during TA muscle degeneration and regeneration may provide another hint (Figure 5F). It was reported that overexpressed CAPN1 suppresses muscle cell differentiation during myogenesis [43]. During the cardiotoxin-induced muscle degeneration, CAPN1's expression decreased, and it recovered during the regeneration process (see Figure 5F), but the absence of CAPN6 delayed the recovery of CAPN1 expression in the regenerating skeletal muscle (compare CAPN1 in Figure 5F lanes 6 and 8). *Capn1* mRNA expression was not changed during this process (Figure 5A), suggesting the involvement of a proteolytic mechanism in this alteration of CAPN1 expression. This CAPN1 change was not observed in primary cultured cell differentiation (Figure 4, data not shown), suggesting an unknown mechanism specific to *in vivo*. At present, however, it cannot be eliminated that the observed CAPN1 change is a result of different muscle conditions caused by *Capn6* disruption. A relationship between CAPN1 and CAPN6 is another interesting subject to be addressed.

In addition, *Capn6* is reported to be overexpressed in the skeletal muscle of patients with limb-girdle muscular dystrophy type 2A (LGMD2A), which is caused by pathological mutations of *CAPN3* [44]. How the physiological role of CAPN6 in skeletal muscle development is related to the pathological condition of muscular dystrophy, where skeletal muscles are in a continuous cycle of degeneration and regeneration, is an intriguing question. In this respect, CAPN6 could be a therapeutic target for muscular dystrophies, since its downregulation is expected to enhance the *de novo* formation of functional skeletal muscles. In terms of tissue engineering, repressing CAPN6 could be helpful for generating skeletal muscle from embryonic or induced pluripotent stem cells *in vitro*.

Despite the predominant expression of CAPN6 during embryogenesis, however, *Capn6*-deficient mice were born and grew healthily, and showed slightly but significantly higher skeletal muscle and body weight (see Figure S3). It is possible that *Capn6* deficiency did not cause severe phenotypes such as lethality or dysgenesis because of a redundancy of CAPN6's non-proteolytic function among calpains. Indeed, recent genetic studies have suggested that calpain family members have non-proteolytic roles in addition to their proteolytic functions [14], [34], [45]. These non-proteolytic functions are ascribed to the CysPc domain, the other domains, or both, and may include the structural regulation of other molecules and/or a chaperone function. Some calpains of schistosomes, insects, and nematodes, and all of the calpains of *Trypanosoma* are non-proteolytic members with substitutions in one or more of the well-conserved active-site triad residues, as seen in eutherian CAPN6 [46]–[49].

A recent bioinformatics analysis revealed that non-classical calpains are considered as the majority among all eukaryotes, and that classical calpains are relatively new in the evolution of calpains, being confined to a small subset of eukaryotes [49]. As mentioned, non-classical calpains contain various non-proteolytic calpains. These findings highlight importance of the calpains' non-proteolytic functions, which are considered to be rather recent functional modifications evolved during calpains' diversification. Although calpains' proteolytic activities are important for understanding many of their physiological functions, recent reports on the non-proteolytic functions of calpains, including this one, shed light on their more elusive physiological roles, and hence may lead to important breakthroughs in biological research and medicine.

## Materials and Methods

### Experimental animals

All procedures using experimental animals were approved by the Experimental Animal Care and Use Committee of Tokyo Metropolitan Institute of Medical Science, and the animals were treated according to the committee's guidelines. C57BL/6 and ICR (a strain established in The Institute of Cancer Research in London, United Kingdom) mice were purchased from Nihon CLEA Inc. For embryos, mice were mated in the evening and if a vaginal plug was observed the next morning, fertilization was assumed to have taken place at midnight. Pregnant mice were sacrificed by cervical dislocation, and the embryos or fetuses were dissected from the uterine decidua.

### Generation of *Capn6*-targeted embryonic stem cells and mutant mice

Genomic clones for mouse *Capn6* were obtained by screening the C57BL6/J-derived BAC libraries from the BACPAC Resource Center. The *nls-lacZ* cassette was made using the *lacZ* gene with a nuclear localization signal (*nls-lacZ*) and flanked with lox71 at the 5′-end and lox2272 at the 3′-end, to allow recombinase-mediated cassette exchange. A pKO Scrambler NTKV-1904 plasmid (Stratagene) was used as the backbone vector. For the targeting construct, a PCR-amplified 1.1-kb fragment and a 5.8-kb NotI-SalI fragment from intron 1 were placed on each side of the *nls-lacZ* cassette. The targeting vector was linearized using NotI and electroporated into the F1 hybrid ES cell line G4 [50]. Clones surviving positive-negative selection with neomycin and FIAU were screened for homologous recombination with diagnostic PCR primers (see Table S1). Of 292 ES cell clones screened, five correctly targeted clones were identified by PCR and confirmed by Southern blot analysis. Three independent ES cell clones hemizygous for the disrupted allele were injected into ICR blastocysts to generate germline chimeras. Female mice homozygous for the *Capn6^lacZ^* allele were obtained by intercrossing F1 hemizygous males (*Capn6^lacZ/Y^*) and heterozygous females (*Capn6^lacZ/+^*). The genotypes of offspring were determined by PCR or Southern blot analysis of tail-tip DNA (Figure 1B). After backcrossing progeny with C57BL6/J mice more than 10 times, the mice were maintained on the B6 genetic background.

### Antibodies and reagents

Polyclonal antibodies against the C- and N-terminal domains of mouse CAPN6 were generated at Transgenic Inc, as described previously [28]. The anti-CAPN6 antibody (ab38940) was purchased from Abcam. An anti-desmin monoclonal antibody (clone DE-U-10), anti-α-sarcomeric actin antibody (clone 5C5), and anti-β-actin monoclonal antibody (clone AC-15) were purchased from Sigma. An anti-myosin heavy chain 3 (Myosin-embryonic clone F1.652) and an anti-myosin heavy chain 8 (Myosin-neonatal clone N1.551) were purchased from Santa Cruz. The anti-CAPN1 monoclonal antibody was a kind gift from Dr. Jiro Takano. Protease inhibitors (E64c, AEBSF, MG-132, PMSF, Calpeptin, and pepstatin A) and chemical reagents were purchased from TaKaRa, Peptide Institute, Sigma, and Kanto Chemical.

### Western blotting

To prepare protein lysates, tissues, and cultured cells were homogenized with TED buffer (10 mM Tris/Cl [pH 7.5], 1 mM EDTA, 1 mM DTT) containing 2% SDS and protease inhibitors, as described above. The protein concentration was determined using a DCA protein assay kit (BioRad), and samples containing equal amounts of protein were separated by 7.5 or 10% SDS-polyacrylamide gel electrophoresis (PAGE) and then electrotransferred to a polyvinylidene difluoride (PVDF) membrane. After being blocked with 5% skim milk in 0.1% Tween 20 in Tris-buffered saline, pH 7.6, the blot was probed with primary antibody. The membrane was then washed with 0.1% Tween 20 in Tris-buffered saline, pH 7.6, and incubated with peroxidase-conjugated anti-rabbit or anti-mouse immunoglobulin G (Dako). The signals were detected using the ECL chemiluminescence detection system (Amersham Bioscience).

### β-Galactosidase staining

*LacZ* expression in *Capn6* mutant mice was detected by staining with X-gal (5-bromo-4-chloro-3-indoyl-β-D-galactoside). Whole embryos were isolated in ice-cold KPP buffer (0.1 M potassium phosphate), then fixed with 4% PFA/0.1% glutaraldehyde in KPP buffer, containing 5 mM EGTA and 2 mM MgCl_2_. The embryos were then rinsed 3 times with wash buffer (0.01% Na deoxycholate and 0.02% Nonidet P-40 in KPP buffer containing 5 mM EGTA and 2 mM MgCl2). Samples for histological analysis were embedded in OTC compound and cryosectioned. The embryos or sections were incubated overnight at 37°C in *lacZ*-staining buffer (10 mM potassium ferrocyanide, 10 mM potassium ferricyanide, and 2 mg/ml X-gal in wash buffer).

### Skeletal muscle primary cell cultures

Mouse skeletal muscle primary cells were prepared as previously described [51], and maintained in F-10 medium containing 10% FBS (fetal bovine serum) and 5 µg/ml FGF-8. To induce muscle differentiation, the medium was switched to differentiation medium (5% horse serum [Invitrogen] and 0.2 M ascorbic acid in Dulbecco's modified Eagle's medium) for further culture. All media were supplemented with 100 units/ml penicillin, 0.1 mg/ml streptomycin, and 2 mM L-glutamine (Invitrogen). To quantify the average number of nuclei per cell, nuclei and cells were labeled by DAPI and rhodamine-phalloidin, respectively, and 100 cells were randomly selected in the fixed area.

### Cardiotoxin injection and tissue preparation

0.1 ml of 10 M cardiotoxin (Wako Pure Chemical Industries) in 0.9% saline was injected directly into the left TA muscle with a 27-gauge needle under ether anesthesia. Mice were killed by cervical dislocation, and the cardiotoxin-injected TA muscles (left) and PBS-injected contralateral TA muscles (right) were removed for analysis. Several of the muscles were frozen in isopentane cooled by liquid nitrogen for histological analysis, and the other muscles were frozen directly in liquid nitrogen for RNA and Protein extraction, and stored at −80°C.

### RT–PCR

Total RNA was extracted using TRIzol reagent (Invitrogen). The reverse transcription reaction was carried out using the First Strand cDNA Synthesis kit (Amersham Bioscience) according to the manufacturer's instructions. The resultant cDNAs were amplified with ExTaq polymerase (Takara) in a thermocycler. The amplified mouse *Capn1* and *Gapdh* were used as internal controls.

### H-E staining and CSA analysis of skeletal muscle

For H-E staining, sections were stained with Mayer's hematoxylin, followed by eosin staining. H-E-stained sections were mounted with Mount-Quick (Daido Sangyo), and viewed with a BX60 microscope (Olympus). The CSA of H-E-stained myofiber sections was determined with NIS-Elements Ar 3.0 software (Nikon).

### Immunofluorescence microscopy

Cells were washed with general tubulin buffer (GTB) (80 mM PIPES [piperazine-N, N′-bis (2-ethanesulfonic acid)], pH 7, 1 mM MgCl_2_, 1 mM EGTA) at room temperature, fixed with 4% paraformaldehyde in GTB, permeabilized with 0.2% Triton X-100 in GTB, and washed with GTB at room temperature. Actin and nuclei were stained with 2.5 units/ml rhodamine-phalloidin and DAPI (Life Technologies), respectively. The cells were viewed using a LSM510 META laser-scanning confocal microscope (Carl Zeiss).

### Statistical analysis

Student's *t*-test was used to test whether or not the means of two normally distributed populations were equal. P values less than 0.05 were considered statistically significant.

## Supporting Information

Figure S1 Additional images of frozen sections of *Capn6^lacZ/lacZ^* (♀) mice with X-gal staining. (A–C) Sections of E17.5 *Capn6^lacZ/lacZ^* embryos. β-Gal expression was detected in intercostal muscle (im), abdominis muscle (am) (A), cartilage primordium of metacarpal bones (B), and orbicularis oculi muscle (om) (C). Scale bars: 500 µm.
(TIF)

Figure S2 Histological analysis of tongue muscle of E16.5 *Capn6^lacZ/+^* (♀) and *Capn6^lacZ/lacZ^* (♀) embryos. (A, B) Low-magnification images of the tongue coronal sections shown in Figure 3A–D. Scale bar: 500 µm. (C) Coronal X-gal-stained sections of the tongue's posterior region. Scale bar: 50 µm. (D) Average diameter of tongue muscle fibers in the posterior region (the areas in boxes a″ and b″; for boxes a′ and b′, see Figure 3E) of coronal sections. The average diameter was significantly larger in *Capn6^lacZ/lacZ^* (7.50 [mean]±0.73 [s.e.m.] µm; n = 5) than in *Capn6^lacZ/+^* (5.46±0.89 µm; n = 5). **, *P*<0.01 by Student's t-test. The results for boxes a′ and b′ are shown in Figure 3E. Het, *Capn6^lacZ/+^* (♀); Ho, *Capn6^lacZ/lacZ^* (♀).
(TIF)

Figure S3 *Capn6* Knockout mice demonstrate promoted muscle growth. (A–D) Comparison of *Capn6^+/Y^* (♂) and *Capn6^lacZ/Y^* (♂) mice at 12 weeks of age. (A) *Capn6^lacZ/Y^* mice are larger than *Capn6^+/Y^* mice. (B) The average body weight of *Capn6^lacZ/Y^* (31.3 [mean]±0.48 [s.e.m.] g; n = 4) was significantly larger than that of *Capn6^+/Y^* (27.1±0.78 g; n = 5). *, P = 0.018 by Student's t-test. (C) Skeletal muscles of *Capn6^lacZ/Y^* mice were larger than those of *Capn6^+/Y^* mice. (D) The average weight of skeletal muscle of *Capn6^lacZ/Y^* (1.50 [mean]±0.056 [s.e.m.] g; n = 4) was significantly larger than that of *Capn6^+/Y^* (1.25±0.027 g; n = 5). **, *P* = 0.004 by Student's t-test. Scale bars: 1 cm. Wt, *Capn6^+/Y^* (♂); Hemi, *Capn6^lacZ/Y^* (♂).
(TIF)

Table S1 List of PCR primers used in this study. Oligonucleotide sequences used in this study for genotyping PCR (upper) and RT-PCR (lower) are listed. For the conditions used for PCR, see the Materials and Methods section.
(DOC)
