# Supplementary figures and images for: Calpain-6 Deficiency Promotes Skeletal Muscle Development and Regeneration
Source: PLoS Genet. 2013 Aug 1;9(8):e1003668. doi: 10.1371/journal.pgen.1003668 (PMC3731218; doi:10.1371/journal.pgen.1003668)

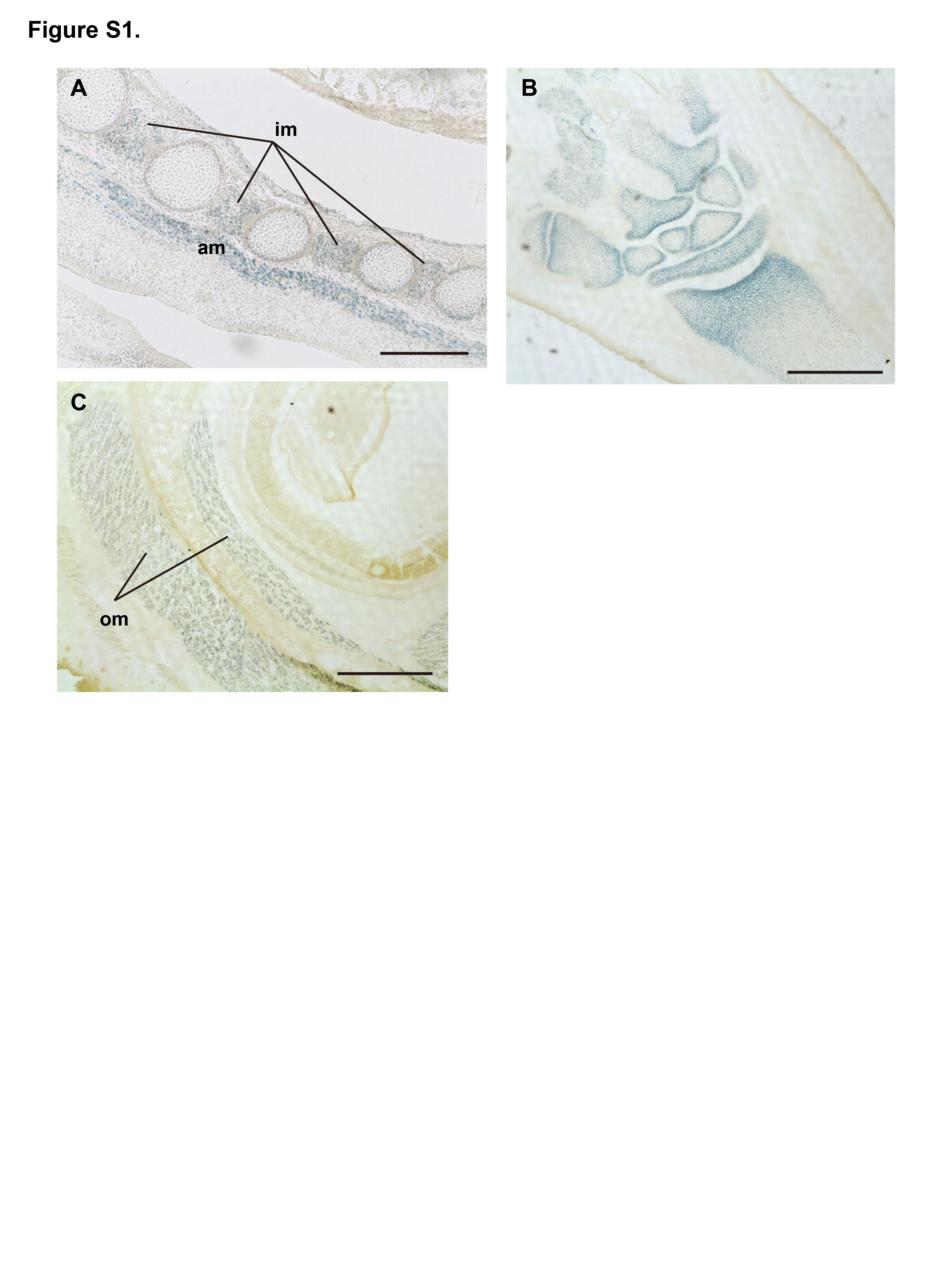

Supplement: Figure S1 — Additional images of frozen sections of Capn6lacZ/lacZ (♀) mice with X-gal staining. (A–C) Sections of E17.5 Capn6lacZ/lacZ embryos. β-Gal expression was detected in intercostal muscle (im), abdominis muscle (am) (A), cartilage primordium of metacarpal bones (B), and orbicularis oculi muscle (om) (C). Scale bars: 500 µm. (TIF) [file pgen.1003668.s001.tif]

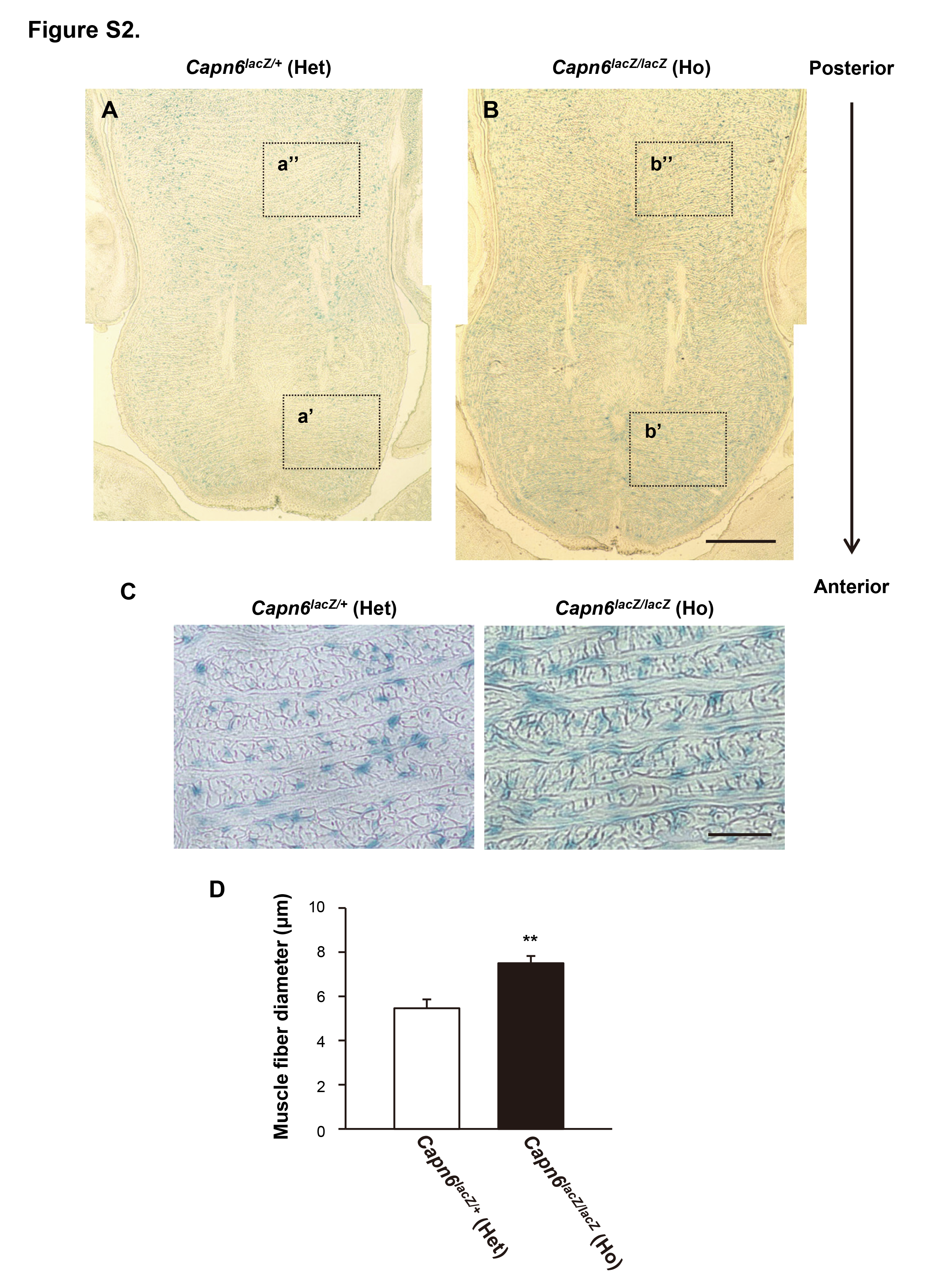

Supplement: Figure S2 — Histological analysis of tongue muscle of E16.5 Capn6lacZ/+ (♀) and Capn6lacZ/lacZ (♀) embryos. (A, B) Low-magnification images of the tongue coronal sections shown in Figure 3A–D. Scale bar: 500 µm. (C) Coronal X-gal-stained sections of the tongue's posterior region. Scale bar: 50 µm. (D) Average diameter of tongue muscle fibers in the posterior region (the areas in boxes a″ and b″; for boxes a′ and b′, see Figure 3E) of coronal sections. The average diameter was significantly larger in Capn6lacZ/lacZ (7.50 [mean]±0.73 [s.e.m.] µm; n = 5) than in Capn6lacZ/+ (5.46±0.89 µm; n = 5). **, P<0.01 by Student's t-test. The results for boxes a′ and b′ are shown in Figure 3E. Het, Capn6lacZ/+ (♀); Ho, Capn6lacZ/lacZ (♀). (TIF) [file pgen.1003668.s002.tif]

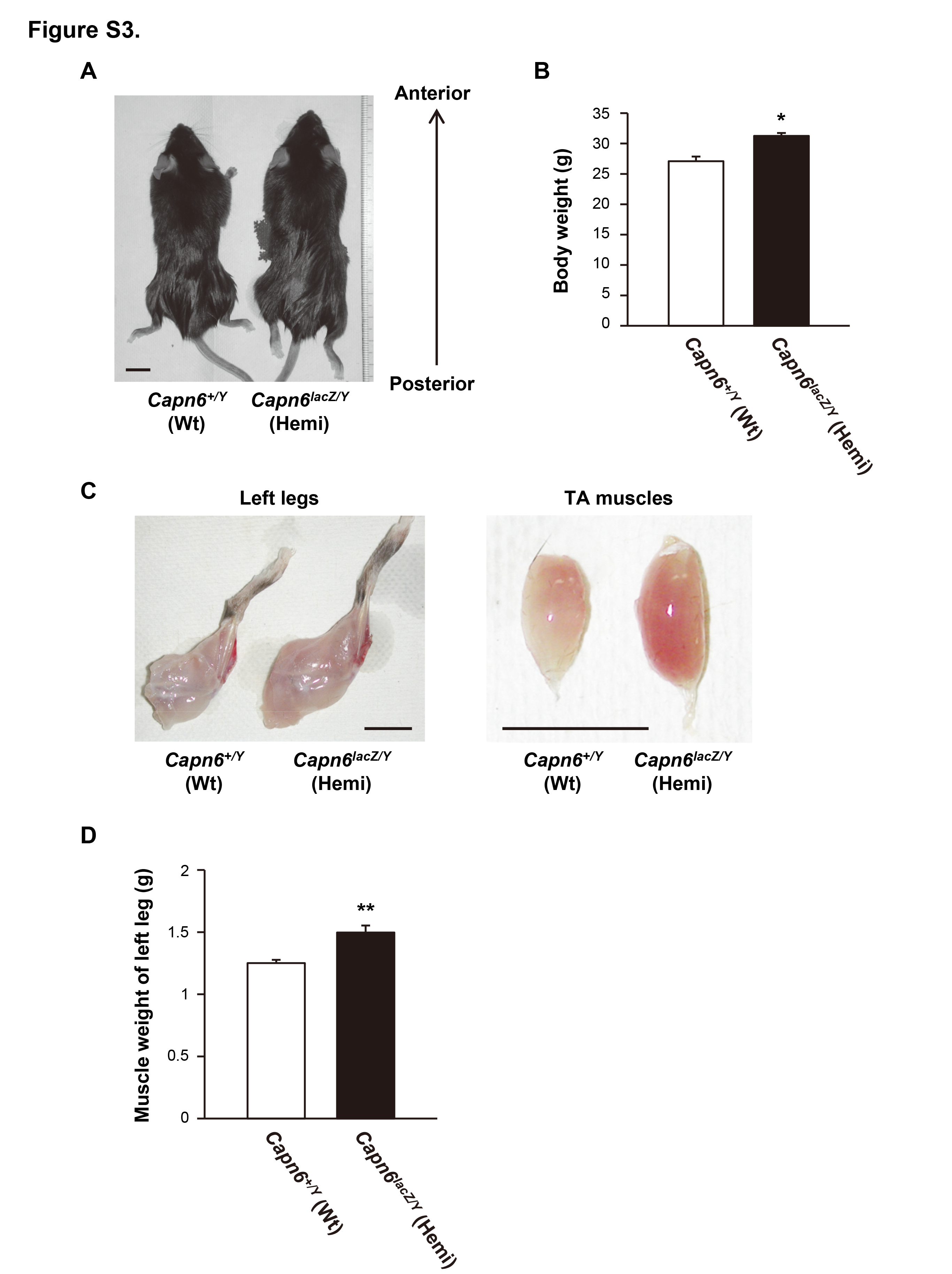

Supplement: Figure S3 — Capn6 Knockout mice demonstrate promoted muscle growth. (A–D) Comparison of Capn6+/Y (♂) and Capn6lacZ/Y (♂) mice at 12 weeks of age. (A) Capn6lacZ/Y mice are larger than Capn6+/Y mice. (B) The average body weight of Capn6lacZ/Y (31.3 [mean]±0.48 [s.e.m.] g; n = 4) was significantly larger than that of Capn6+/Y (27.1±0.78 g; n = 5). *, P = 0.018 by Student's t-test. (C) Skeletal muscles of Capn6lacZ/Y mice were larger than those of Capn6+/Y mice. (D) The average weight of skeletal muscle of Capn6lacZ/Y (1.50 [mean]±0.056 [s.e.m.] g; n = 4) was significantly larger than that of Capn6+/Y (1.25±0.027 g; n = 5). **, P = 0.004 by Student's t-test. Scale bars: 1 cm. Wt, Capn6+/Y (♂); Hemi, Capn6lacZ/Y (♂). (TIF) [file pgen.1003668.s003.tif]
