# Supplementary material for: Calpain-6 Deficiency Promotes Skeletal Muscle Development and Regeneration
Source: PLoS Genet. 2013 Aug 1;9(8):e1003668. doi: 10.1371/journal.pgen.1003668 (PMC3731218; doi:10.1371/journal.pgen.1003668)
Supplement: Table S1 — List of PCR primers used in this study. Oligonucleotide sequences used in this study for genotyping PCR (upper) and RT-PCR (lower) are listed. For the conditions used for PCR, see the Materials and Methods section. (DOC) [file pgen.1003668.s004.doc]

**Supplementary Table**

**Supplementary Table S1.** **List of PCR primers used in this study.**

Oligonucleotide sequences used in this study for genotyping PCR (upper) and RT-PCR (lower) are listed. For the conditions used for PCR, see the Materials and Methods section.

Genotyping PCR

| Primers | | Sequence |
| --- | --- | --- |
| WT | sense | 5'-GCTCTCTACCACGATCCTTTATCC-3' |
| Neo | sense | 5'-CCACTCCCACTGTCCTTTCCTAAT-3' |
| Rev | antisense | 5'-TGTATGTTTTCAATGCTCCCCTGG-3' |

RT-PCR

| Primers | | Sequence | Accession No. |
| --- | --- | --- | --- |
| Capn6 mRNA | sense | 5'-GAATTCATGGGTCCTCCTCTGAAGCT-3' | NM_007603 |
| (full length 1925 bp) | antisense | 5'-GAATTCGAGCTCAGTGAGATCATCGC-3' |  |
| Myh3 mRNA | sense | 5'-ACACCAAGAAGAAGTTGGAG-3' | NM_001099635 |
| (771 bp) | antisense | 5'-CCAACTTGAAACAAGGCAAA-3' |  |
| Myh8 mRNA | sense | 5’-GATGTTTCACAGCTGCAGAG-3’ | NM_177369 |
| (727 bp) | antisense | 5’-GTCAGCAGTAGGAGAAAAGT-3’ |  |
| Capn1 mRNA | sense | 5'-ACTCGAGCCCCAGGATGACAGAGGA-3' | NM_007600 |
| (full length 2141 bp) | antisense | 5'-AGAATTCTCAGGCAAACATAGTCAGCTG-3' |  |
| Gapdh mRNA | sense | 5'-GGTGTGAACCACGAGAAATAT-3' | NM_008084 |
| (315 bp) | antisense | 5'-AGATCCACGACGGACACATT-3' |  |
